# Increased amyloidogenic processing of transgenic human APP in X11-like deficient mouse brain

**DOI:** 10.1186/1750-1326-5-35

**Published:** 2010-09-15

**Authors:** Maho Kondo, Maki Shiono, Genzo Itoh, Norio Takei, Takahide Matsushima, Masahiro Maeda, Hidenori Taru, Saori Hata, Tohru Yamamoto, Yuhki Saito, Toshiharu Suzuki

**Affiliations:** 1 Laboratory of Neuroscience, Graduate School of Pharmaceutical Sciences, Hokkaido University, Kita12-Nishi6, Kita-ku, Sapporo 060-0812, Japan; 2 Immuno-Biological Laboratories Co. Ltd. (IBL), Mikasa Laboratory, Okayama 440-22, Mikasa 068-2165, Japan; 3 Immuno-Biological Laboratories Co. Ltd. (IBL), Naka 1091-1, Fujioka 375-0005, Japan; 4 Creative Research Institute Sousei, Hokkaido University, Kita21-Nishi10, Kita-ku, Sapporo 011-0021, Japan; 5 Laboratory of Neuronal Cell Biology, Graduate School of Pharmaceutical Sciences, Hokkaido University, Kita12-Nishi6, Kita-ku, Sapporo 060-0812, Japan

## Abstract

**Background:**

X11-family proteins, including X11, X11-like (X11L) and X11-like 2 (X11L2), bind to the cytoplasmic domain of amyloid β-protein precursor (APP) and regulate APP metabolism. Both X11 and X11L are expressed specifically in brain, while X11L2 is expressed ubiquitously. X11L is predominantly expressed in excitatory neurons, in contrast to X11, which is strongly expressed in inhibitory neurons. *In vivo *gene-knockout studies targeting X11, X11L, or both, and studies of X11 or X11L transgenic mice have reported that X11-family proteins suppress the amyloidogenic processing of endogenous mouse APP and ectopic human APP with one exception: knockout of X11, X11L or X11L2 has been found to suppress amyloidogenic metabolism in transgenic mice overexpressing the human Swedish mutant APP (APPswe) and the mutant human PS1, which lacks exon 9 (PS1dE9). Therefore, the data on X11-family protein function in transgenic human APP metabolism *in vivo *are inconsistent.

**Results:**

To confirm the interaction of X11L with human APP ectopically expressed in mouse brain, we examined the amyloidogenic metabolism of human APP in two lines of human APP transgenic mice generated to also lack X11L. In agreement with previous reports from our lab and others, we found that the amyloidogenic metabolism of human APP increased in the absence of X11L.

**Conclusion:**

X11L appears to aid in the suppression of amyloidogenic processing of human APP in brain *in vivo*, as has been demonstrated by previous studies using several human APP transgenic lines with various genetic backgrounds. X11L appears to regulate human APP in a manner similar to that seen in endogenous mouse APP metabolism.

## Background

X11 proteins (X11s) comprise a family of three adaptor proteins in mammals: X11 (X11/X11α/Mint1), X11-like (X11L/X11β/Mint2) and X11-like 2 (X11L2/X11γ/Mint3) [1]. These molecules are evolutionally conserved in *D. melanogaster *[2,3] and *C. elegans *[4]. In mammals, X11 and X11L are expressed predominantly in neurons, while X11L2 is expressed ubiquitously [reviewed in ref. [5]]. X11s associate with the cytoplasmic domain of amyloid β-protein precursor (APP) and suppress APP metabolism, including amyloid β-protein (Aβ) generation [1,6,7], which is widely believed to be the major cause of Alzheimer's disease (AD) pathogenesis [8]. APP is subjected to alternative cleavages by a combination of α- and γ-secretases or β- and γ-secretases. Primary cleavage of APP by α-secretase is amyloidolytic and generates a C-terminal fragment, CTFα, which includes the C-terminal half of the Aβ sequence, whereas cleavage by β-secretase is amyloidogenic and generates CTFβ, which includes an intact Aβ sequence. Both CTFα and CTFβ are further cleaved by γ-secretase in the lipid bilayer, resulting in the secretion of the amyloidolytic p3 fragment from CTFα and the neurotoxic Aβ from CTFβ [9].

The association of X11s with APP is mediated by interaction between the phosphotyrosine interaction domain (PTB) of X11s and the 681-GYENPTY-687 motif of APP. This association has been shown to greatly suppress the amyloidogenic metabolism of APP in the brain *in vivo*. Specifically, the production of amyloidogenic CTFβ, but not amyloidolytic CTFα, derived from endogenous mouse APP was found to be enhanced, and accumulation of mouse Aβ was found to be increased, in the brains of X11-knockout, X11L-knockout, and X11 plus X11L double-gene knockout mice [10-12]. Recent evidence indicates that the majority of both β- and γ-secretases are located in cholesterol- and sphingolipid-rich detergent-resistant membrane domains (DRM domains or lipid rafts) as active forms [13,14]. DRMs in the brains of mice lacking X11 and X11L are rich in mature APP (*N- *and *O-*glycosylated form), the substrate to secretases, and the amyloidogenic metabolite CTFβ [11]. These observations suggest that X11 and X11L function to form a complex with APP that then remains outside of the DRMs. In this way, they regulate amyloidogenic cleavage of APP through suppression. This suppressive inhibition of human APP amyloidogenic metabolism by X11 and X11L was also confirmed in the brains of X11- or X11L-Tg mice expressing human Swedish mutant APP (APPswe/Tg2576) [15,16] (Table 1). On the other hand, a controversial report analyzing human APP metabolism in the brains of mice lacking X11s found that the amyloidogenic metabolism of APPswe was suppressed in the brains of mice lacking X11 proteins and constitutively expressing the active PS1 mutant PS1dE9 [17] (Table 1). In this study, we investigated whether the amyloidogenic metabolism of human APP, as well as endogenous mouse APP, is facilitated in murine brains lacking X11L. We focused on X11L, rather than X11, because it is more widely distributed and because it suppresses APP metabolism more strongly [10,11]. Our data showed increased amyloidogenic metabolism in two transgenic mouse lines expressing relatively higher (APP23) and lower (APP-ibl) levels of human APPswe in the absence of X11L, indicating that X11L functions to suppress APP amyloidogenic metabolism in brain *in vivo*.

**Table 1:** Effect of X11s in the generation of Aβ in the brains of several transgenic and knock-out mouse lines

Reference number in text	Authors & Journal	X11 family genes	APP and PS genes	Examination of brain Aβ levels	Effect of X11s in the generation of Aβ
[10]	Sano *et al*., *J. Biol. Chem*. (2006) 281, 37853-37860.	X11L, knock-out	endogenous mouse APP	mouse Aβ40 mouse Aβ42	**suppressive**
[11]	Saito *et al*., *J. Biol. Chem. *(2008) 283, 35763-35771	X11, X11L, & X11/X11L, knock-out	endogenous mouse APP	mouse Aβ40 mouse Aβ42	**suppressive**
[15]	Lee *et al*., *J. Biol. Chem*. (2003) 278, 47025-47029.	X11, transgenic	human APPswe transgenic (Tg2576)	human Aβ40 human Aβ42	**suppressive**
[16]	Lee *et al*., *J. Biol. Chem*. (2004) 279, 49099-49104.	X11L, transgenic	human APPswe transgenic (Tg2576)	human Aβ40 human Aβ42	**suppressive**
[17]	Ho *et al*., *J. Neurosci*. (2008) 28, 14392-14400.	X11, X11L or X11L2, knock-out	human APPswe/PS1dE9 double-transgenic	human Aβ40 human Aβ42	**facilitative**
[23]	Saluja *et al*., *Neurobiol. Dis*. (2009) 36, 162-168.	X11, heterozygous knock-out	human APPswe/PS1dE9 double-transgenic	human Aβ40 human Aβ42	**suppressive**
[24]	Mitchell *et al*., *Hum. Mol. Genet. *(2009) 18, 4492-4500.	X11L, transgenic	human APPswe transgenic (Tg2576)	human Aβ40 human Aβ42	**suppressive**
**Present results**	Kondo *et al*., *Mol. Neurodegen*.	X11L, knock-out	human APPswe transgenic (APP23), human APPswe transgenic (APP-ibl)	human Aβ40 human Aβ42	**suppressive**

## Results

### APP metabolism in APP23 mouse brain lacking X11L

The amyloidogenic metabolism of endogenous APP in brain was facilitated in X11-, X11L-, and X11 plus X11L- gene knockout mice, while a contrary result was reported for exogenously expressed human APPswe in X11s (X11, X11L or X11L2)- gene knockout mice [17]. X11L-gene knockout mice have been shown to exhibit more strongly enhanced amyloidogenic metabolism of endogenous APP as compared with X11-gene knockout mice [10,11]. Thus, we reexamined the function of X11L in the suppression of APP amyloidogenic metabolism using X11L-gene knockout mice and human APPswe transgenic mouse lines.

**Figure 1:**
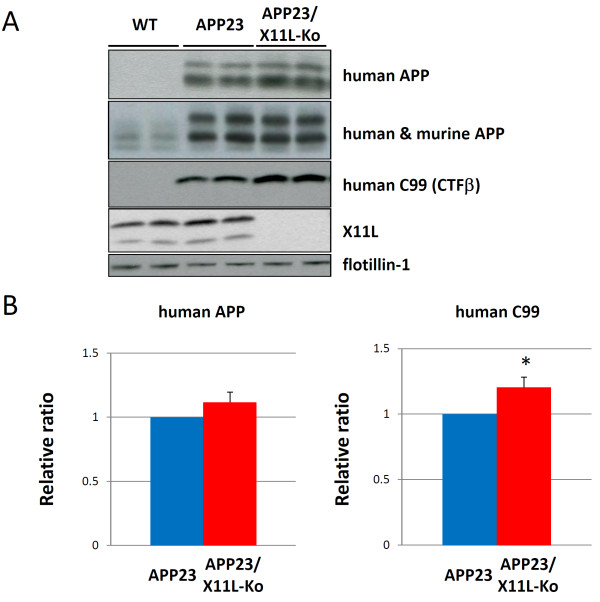
**Expression of APP and generation of primary amyloidogenic fragment CTFβ in APP23 mice in the presence or absence of X11L**. (**A**) Expression of human APP751swe and detection of human CTFβ (C99) in APP23 mouse brain samples in the presence or absence of X11L and in wild-type mice. Membrane (P100 for APP, CTFβ and flotillin-1) and cytoplasmic (S100 for X11L) fractions of brain regions composed of the cerebral cortex, hippocampus and olfactory bulb from wild-type (WT), APP23 and APP23/X11L-Ko mice (5-6 months old) were analyzed for the expression of transgenic human APP751swe (anti-APP 10D1 antibody), total APP (human APP751swe combined with mouse endogenous APP695, anti-APP/c 8717 antibody), human APP C99 (CTFβ, anti-human Aβ 82E1 antibody), X11L and flotilin-1 by immunoblotting. For human APP, the upper band indicates mature APP751swe (mAPP) and the lower band indicates immature APP751swe (imAPP). For mouse APP (see lanes of WT), the upper two bands are mAPP695 and lower band is imAPP695. To identify CTF species with or without phosphorylation at Thr668, the lysates were subjected to dephosphorylation prior to detection as described in *Materials and Methods *(for identification of APP and CTF molecules, reviewed in Suzuki and Nakaya [12]). (**B**) The densities of human APP751swe (left) and C99 (right) bands were standardized to the density of flotillin-1, which was compared with the ratios for APP23 mice that were assigned a reference value of 1.0 (values represent the means ± S.E.). The data were analyzed by Student's t test (n = 10; *, p < 0.05).

We first generated APP23 mice lacking X11L because APP23 mice have been established as a transgenic line expressing human APP751swe [18,19], and the expression level of APP in these mice is high enough to allow the observation of amyloid plaques in mice at around 10 months. The overproduction of Aβ is still milder in APP23 than in APPswe-Tg/PS1dE9-Tg, another human APP transgenic mouse model that constitutively overproduces Aβ to grow amyloid plaques at younger ages and has been used in previous analyses [17,20]. APP23 may be the better model for precise evaluation of the regulatory role of X11L. We first examined human APPswe expression in the APP23 X11L knockout (APP23/X11L-Ko) mouse brain. The brain regions used included the cerebral cortex, hippocampus and olfactory bulb of wild-type, APP23 and APP23/X11L-Ko mice (5-6 months old). Membrane (P100) and cytoplasmic (S100) fractions were prepared and analyzed for human APP, human-plus-mouse APP, X11L and flotillin-1 (as a marker for membrane protein) by immunoblotting with respective antibodies (Fig. 1A). We confirmed that the human APP level did not change significantly in APP23 mice lacking X11L (Fig. 1A, **first rows; Fig**. 1B, **left**). Using the same samples, we examined the level of amyloidogenic fragment (CTFβ/C99) derived from human APP by immunoblotting with anti-human, Aβ-specific antibody (Fig. 1A, **third rows**). Our previous results indicated that CTFβ (C99) derived from endogenous mouse APP increased in X11L-deficient mouse brains [11]. In agreement with these data, we found that the human CTFβ (C99) from APPswe also increased significantly in the brains of APP23/X11L-Ko mice when compared with APP23 mice (Fig. 1A, **third rows; Fig**. 1B, **right**).

**Figure 2:**
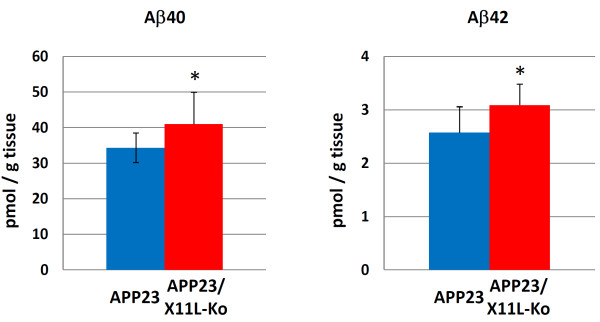
**Human Aβ levels in APP23 mouse brain samples in the presence or absence of X11L**. Quantification of human Aβ40 (left) and Aβ42 (right) in brain samples composed of the cerebral cortex, hippocampus and olfactory bulb from APP23 and APP23/X11L-Ko mice (5-6 months old) was performed by sELISA. Concentrations were normalized to tissue weight. The data were analyzed by Student's t test. Statistical significance is indicated with asterisks (n = 10; *, p < 0.05). The error bars are S.E.

**Figure 3:**
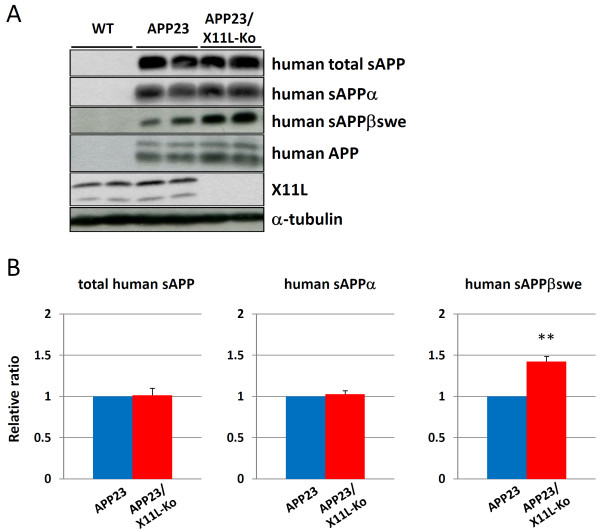
**Human sAPP levels in APP23 mouse brain samples in the presence or absence of X11L**. **(A) **Detection of total human sAPP, sAPPα and sAPPβswe along with human APP, X11L and α-tubulin in APP23 mouse brain tissue in the presence or absence of X11L. Membrane (P100 for APP) and cytoplasmic (S100 for sAPP, sAPPα, sAPPβswe, X11L and α-tubulin) fractions of brain regions composed of cerebral cortex, hippocampus and olfactory bulb from wild-type (WT), APP23 and APP23/X11L-Ko mice (5-6 months old) were analyzed for total human sAPP (sAPPα plus sAPPβswe by 10D1 antibody), human sAPPα (2B3 antibody), human sAPPβswe (6A1 antibody), human APP (10D1 antibody), X11L and α-tubulin by immunoblotting. (**B**) The densities of total human sAPP (left), sAPPα (middle) and sAPPβswe (right) bands were standardized to the density of α-tubulin, which was compared with the ratios for APP23 mice that were assigned a reference value of 1.0 (values represent the means ± S.E.). The data were analyzed by Student's t test (n = 10; **, p < 0.01).

We further examined secreted human Aβ and human sAPP, large extracellular N-terminal domain truncated at the α-site (sAPPα) and/or β-site (sAPPβ), levels in the same samples (Figs. 2 and 3). Human Aβ40 and Aβ42 significantly increased in APP23/X11L-Ko mice (Fig. 2). Since the difference between Aβ40 and Aβ42 is dependent on alternative intramembrane γ-cleavage, we investigated the increase in primary β-site cleavage by detecting human sAPPβswe (Fig. 3A, **third rows**). The sAPPβswe, derived from human APPswe, was detected by immunoblotting with anti-sAPPβswe specific antibody, which showed a significant increase in sAPPβswe in APP23/X11L-Ko mice (Fig. 3B, **right**). The total levels of human sAPP and sAPPα were not significantly altered (Fig. 3A, **first and second rows; Fig**. 3B, **left and middle**). These analyses indicate that a lack of X11L facilitates the primary amyloidogenic processing of human APP at the β-site, but not at the α-site, as has been previously shown for the processing of endogenous mouse APP in X11L-Ko mouse brains [11].

**Figure 4:**
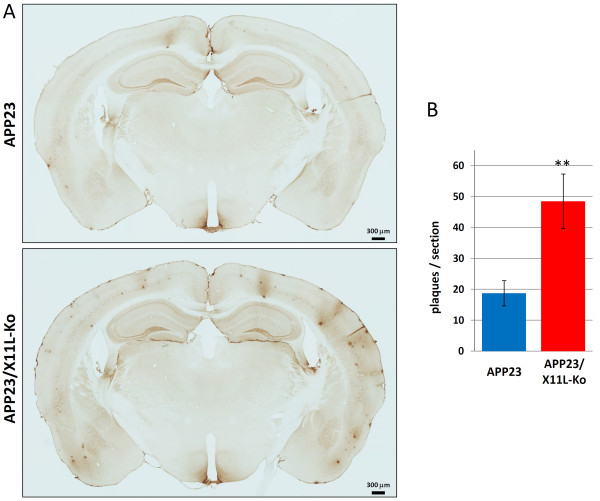
**Quantification of amyloid plaques in APP23 mouse brain in the presence or absence of X11L**. Immunostaining of coronal sections of the brain region including the cerebral cortex and hippocampus of APP23 (Fig. 4A, upper panels) and APP23/X11L-Ko mice (Fig. 4B, lower panels) at 12 months of age is shown. The brain sections were stained with anti-Aβ 82E1 antibody to detect human Aβ. Stains indicate amyloid plaque. Bar, 300 μm. The data were analyzed by Student's t test (n = 4 serial sections × 6 individuals respectively; **, p < 0.01). The error bars are S.E.

If amyloidogenic processing of human APP were to progress in the brains of APP23/X11L-Ko mice, amyloid plaques would be expected to appear at younger ages than is seen in APP23 mice. It has been well established that amyloid plaque formation appears in ~10 month APP23 mice. Thus, we examined the formation of amyloid plaques in both 5, 9 and 12 month mice of APP23 and APP23/X11L-Ko. At five months, amyloid plaques appeared in neither APP23, nor APP23/X11L mice (Additional file 1, **Fig. S1**), but at nine months, amyloid plaques appeared in both APP23 and APP23/X11L-Ko mice (Additional file 1, **Fig. S2**). Brain slices of mice (12 months old) were immunostained with anti-human Aβ antibody, the plaque numbers increased remarkably in APP23/X11L-Ko when compared to APP23 (Fig. 4A). The numbers of amyloid plaques in the two lines, APP23 and APP23/X11L-Ko (12 months old), were quantified in 18 serial sections from six individuals (Fig. 4B). The sections, including the cerebral cortex, the hippocampus and the entorhinal cortex, were analyzed for the plaque numbers. The average number of amyloid plaques was 48.5 per section in APP23/X11L-Ko mice, but only 18.8 per section in APP23 mice, indicating that plaque numbers significantly increase in brains lacking X11L.

Taken together with previous observations that the numbers of amyloid plaques were decreased in the brains of X11- or X11L-tg mice expressing human APPswe [15,16], the present observation indicates that X11L plays an important role in the suppression of the amyloidogenic and pathogenic metabolism of human APP, as well as the observation of facilitated amyloidogenic metabolism of endogenous APP in X11-, X11L-, and X11 plus X11L- knockout mice [10,11].

### APP metabolism in APP-ibl mouse brain lacking X11L

**Figure 5:**
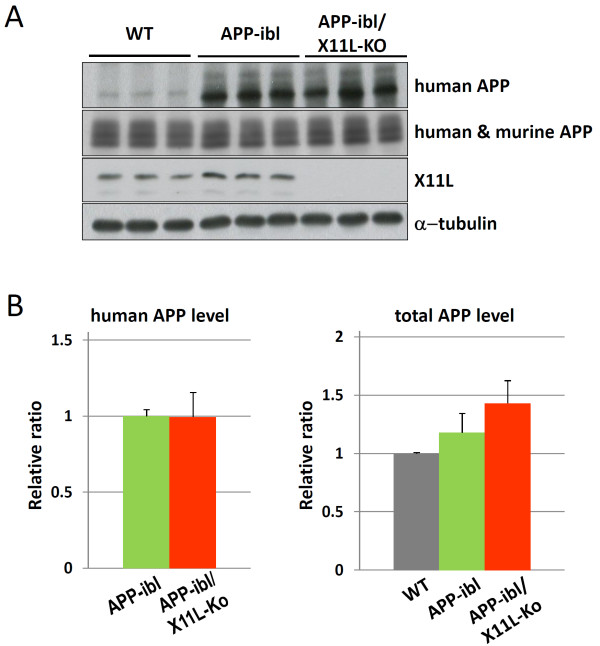
**Expression of APP in APP-ibl mouse brain in the presence or absence of X11L**. (**A**) Expression of human APP695swe and mouse endogenous APP695. Brain regions composed of the cerebral cortex, hippocampus and olfactory bulb from wild-type (WT), APP-ibl and APP-ibl/X11L-Ko mice (4 months old) were homogenized in 10 mM Tris-HCl [pH 7.9] containing 1% (w/v) SDS, 4 M urea, complete protease inhibitor cocktail (Roche Diagnostics), and 1 μM pepstatin. The lysates were analyzed by immunoblotting with anti-human APP (10D1), anti-APP (8717), anti-X11L and anti-α-tubulin antibodies. (**B**) The densities of human APP695swe (left) and human plus mouse APP (right) bands were standardized to the density of α-tubulin, which was compared with the ratios for APP-ibl mice (left) or wild-type mice (right), which were assigned a reference value of 1.0 (values represent means ± S.E.). The data were analyzed by Student's t test (n = 3).

**Figure 6:**
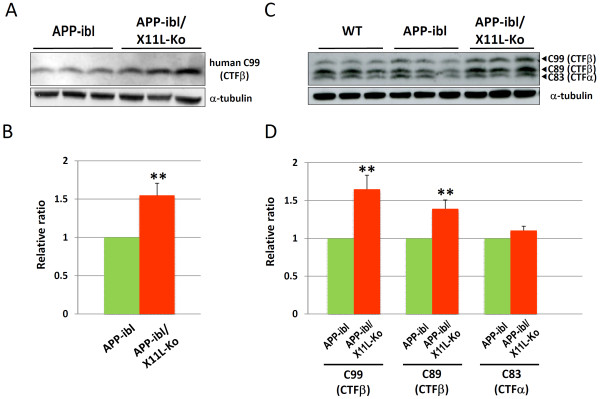
**Generation of primary amyloidogenic fragment CTFβ in APP-ibl mice in the presence or absence of X11L**. (**A**) Generation of human CTFβ in APP-ibl mice in the presence or absence of X11L. Brain regions composed of the cerebral cortex, hippocampus and olfactory bulb from APP-ibl and APP-ibl/X11L-Ko mice (4 months old) were homogenized in 10 mM Tris-HCl [pH 7.9] containing 1% (w/v) SDS, 4 M urea, complete protease inhibitor cocktail (Roche Diagnostics), and 1 μM pepstatin. The lysates were dephosphorylated as described [11,27] and analyzed by immunoblotting with anti-human Aβ (82E1) and anti-α-tubulin antibodies. (**B**) The density of human CTFβ band was standardized to the density of α-tubulin, which was compared with the ratios for APP-ibl mice that were assigned a reference value of 1.0 (values represent the means ± S.E.). The data were analyzed by Student's t test (n = 10; **, p < 0.01). (**C**) Generation of CTFα and CTFβ in APP-ibl mice in the presence or absence of X11L. Brain lysates of the cerebral cortex, hippocampus and olfactory bulb from wild-type (WT), APP-ibl and APP-ibl/X11L-Ko mice (4 months old) were dephosphorylated and analyzed by immunoblotting with anti-APP/c 8717 and anti-α-tubulin antibodies. (**D**) The densities of C99 (CTFβ), C89 (CTFβ) and C83 (CTFα) bands were standardized to the density of α-tubulin, which was compared with the ratios for APP-ibl mice that were assigned a reference value of 1.0 (values represent means ± S.E.). The data were analyzed by Student's t test (n = 10; **, p < 0.01).

**Figure 7:**
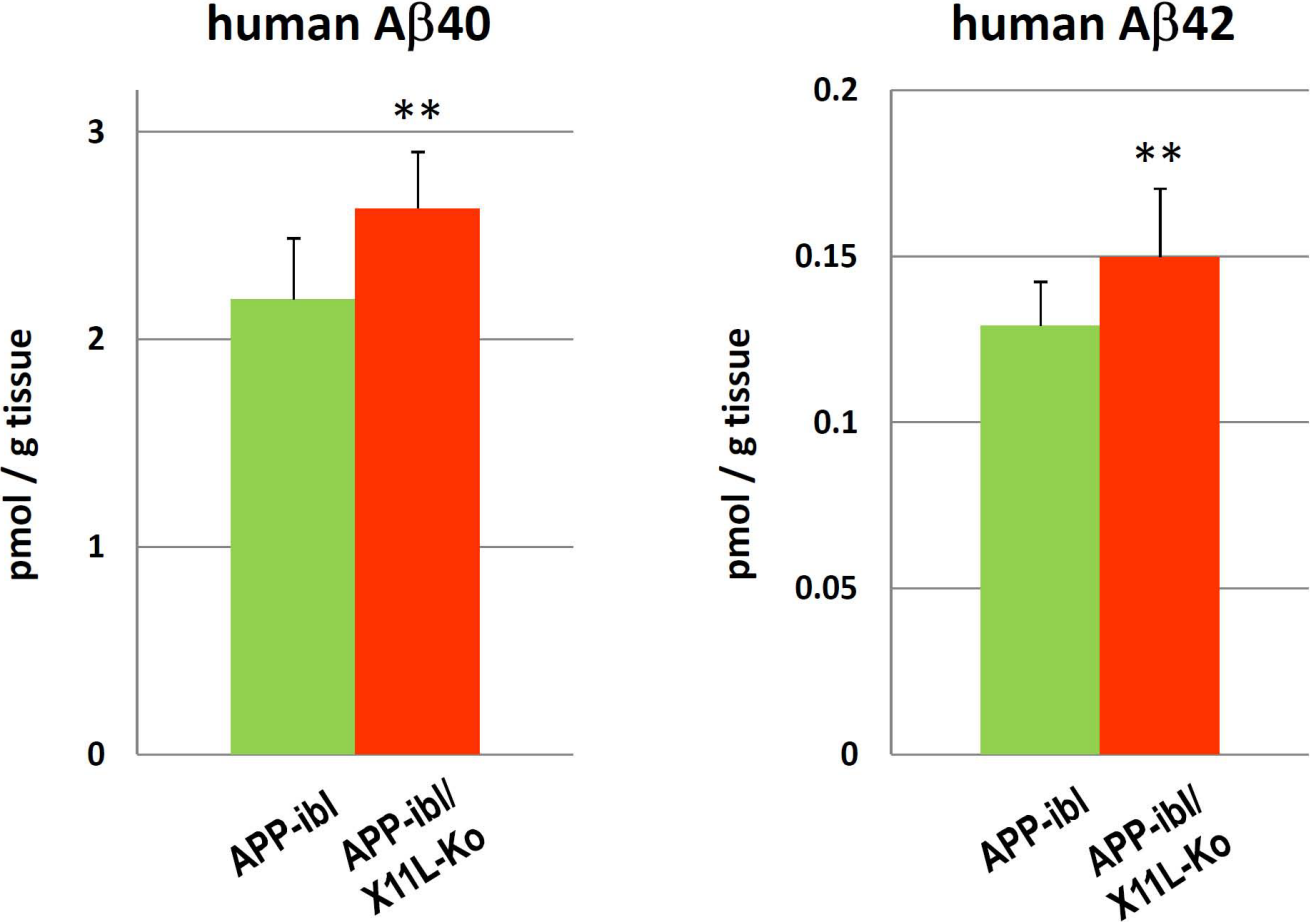
**Human Aβ levels in APP-ibl mouse brain in the presence or absence of X11L**. Quantification of human Aβ40 (left) and Aβ42 (right) in the cerebral cortex, hippocampus and olfactory bulb from APP-ibl and APP-ibl/X11L-Ko mice (5-6 months old) was performed by sELISA, with concentrations normalized to tissue weight. The data were analyzed by Student's t test. Statistical significance is indicated with asterisks (n = 10; **, p < 0.01). The error bars are S.E.

The analysis described above was also performed with another APP transgenic mouse line, APP-ibl, which was generated using human APP695swe. In contrast to APP23, which expressed relatively higher levels of human APP751swe, APP-ibl expressed levels of human APP 1.2-1.4 times those of endogenous mouse APP (Fig. 5A, **second rows; Fig**. 5B, **right**). The human APP level was similar between APP-ibl and APP-ibl/X11L-Ko mice brain (Fig. 5A, **first rows; Fig**. 5B, **left**), as was observed in APP23 and APP23/X11L-Ko (Fig. 1). The level of the amyloidogenic fragment of human APP, CTFβ (C99), increased significantly in APP-ibl/X11L-Ko (5-6 months old) when compared to the same age of APP-ibl mice (Fig. 6A**and **6B). We also examined the levels of another CTFβ (C89) and CTFα (C83), along with CTFβ (C99), by immunoblotting with pan-APP CTF antibody, which detects both human and mouse CTFs (Fig. 6C**and **6D). Both C99 and C89 CTFβ levels increased significantly in APP-ibl/X11L-Ko mouse brains, while the levels of CTFα (C83) were the same regardless of X11L expression. The enhanced amyloidogenic metabolism of APP was further confirmed by quantifying brain Aβ levels, which increased significantly in APP-ibl/X11L-Ko as compared with APP-ibl mice for both Aβ40 and Aβ42 at 5-6 months of age (Fig. 7). In contrast to the APP23 mice, at 12 months of age, the APP-ibl mice had not generated any amyloid plaques (data not shown). This is probably because the levels of Aβ40 and Aβ42 are much lower than those in APP23 mice (1/15-1/20 the levels of APP23 mice) (compare Fig. 2 to Fig. 7).

In summary, we used two human APP transgenic mouse lines lacking the X11L gene and found that X11L functions in the suppression of amyloidogenic metabolism of human APP, as it does for mouse endogenous APP, in brain *in vivo*.

## Discussion

The qualitative and quantitative alteration of Aβ generation is a major cause of AD pathogenesis. Familial Alzheimer's disease (FAD)-linked presenilin-1 mutations increase the longer, pathogenic Aβ42 species. APP locus duplication also induces an increase in Aβ generation. Both of these cases, one showing qualitative and one showing quantitative alteration of Aβ generation, induce early-onset AD [8,21,22]. Because the pathological progression of sporadic AD (SAD) is similar to that of FAD, the regulation of Aβ generation (in terms of both quality and quantity) is also important in SAD pathogenesis, regardless of the absence of known causative genetic mutations.

The regulation of APP metabolism is closely related to intracellular protein sorting, in which many regulatory molecules associate directly or indirectly with APP [9,12]. The X11 family proteins X11, X11L and X11L2 directly associate with APP through their PTB domains. This protein interaction is thought to regulate the metabolism and intracellular trafficking of APP. In cells expressing APP together with X11, X11L, or X11L2, APP metabolism is remarkably suppressed, with a consequential decrease in Aβ generation [1,6,7]. However, *in vitro *studies have not been able to fully characterize the molecular function of X11L in APP metabolism in brain. To resolve this issue, several lines of transgenic and knockout mice for X11 genes were produced and used to examine APP metabolism in brain *in vivo*, including Aβ generation and/or amyloid plaque formation. X11 and X11L transgenic mice expressing human APPswe show decreased levels of cerebral Aβ and reductions in Aβ plaques in comparison with mice expressing APPswe alone [15,16]. Furthermore, amyloidogenic metabolism of endogenous mouse APP has been shown to be facilitated in the brains of X11-, X11L- and X11 plus X11L- gene knockout mice [10,11], indicating that X11s function physiologically to suppress the amyloidogenic metabolism of APP. These results have been confirmed by similar studies [23,24], but also conflict with a report that X11s (X11, X11L or X11L2)- gene Ko mice overexpressing APPswe and PS1dE9 decreased Aβ generation at younger ages [17].

We consider that the contrary results may be dependent on the presence or absence of mutations in the PS1 gene because PS1 is known to regulate intracellular protein trafficking [25]. Transgenic mice with APPswe and PS1dE9 genes (APPswe/PS1dE9-tg) generate larger amounts of Aβ without intracellular regulation of APP metabolism and trafficking. APPsw/PS1dE9 mice are suitable as an AD model showing AD pathology [20], but may not be suitable for analysis of the regulation of intracellular APP metabolism. Therefore, in this study, we used two lines of X11L-Ko mice expressing human APP. One is an X11L-Ko line based on APP23 that expresses APP751swe [19]. The other, APP-ibl, is an X11L-Ko line expressing APP695swe in lower levels. We used X11L-Ko mice alone to examine the effects on the metabolism of the transgenic APP molecule because X11L-Ko mice showed a stronger increase in amyloidogenic metabolism of endogenous APP than X11-Ko. The level of this effect was similar to that in X11/X11L double-Ko mice [11]. We confirmed that, in both mice expressing higher (APP23) and lower (APP-ibl) levels of APPswe in the absence of X11L, amyloidogenic APP metabolism increased. These results coincide well with previous reports demonstrating that X11s function to suppress [10,11,15,16,23,24], but not to enhance [17], the amyloidogenic metabolism of APP (summarized in Table 1).

## Conclusions

In conclusion, X11L plays an important role in suppressive regulation of APP amyloidogenic metabolism in brain *in vivo*. The metabolic regulation of APP by X11s may provide useful targets in the development of drugs to suppress the amyloidogenic metabolism of APP in AD.

## Materials and methods

### Mice

The X11L-Ko mouse has been described [10]. The human APP751swe-tg APP23 mouse was kindly supplied from Novartis Pharma Inc. [19]. The APP-ibl transgenic mouse was generated through transduction of human APP695swe cDNA driven by the PDGFβ promotor. The DNA was injected into fertilized eggs from a BDF strain mouse, and the founder was selected by DNA hybridization and then subjected to back-cross with the C57BL/6 strain. APP23/X11L-Ko and APP-ibl/X11L-Ko mice were generated by mating with X11L-Ko mice generated from a C57BL/6 background [10], and heterozygous human APPswe transgenic [tg+/-, X11L-/-] and [tg+/-, X11L+/+] mutant mice were used for the study.

### Antibodies

Mouse monoclonal antibodies to human Aβ 82E1 (IBL) and 6E10 (Signet COVANCE), human APP 10D1 (IBL), sAPPα 2B3(IBL), sAPPβswe 6A1 (IBL), actin (Chemicon), flotillin-1 (BD Transduction Laboratories), α-tubulin (Zymed and Santa Cruz Biotechnologies), and X11L/Mint2 (BD Transduction Laboratories) were purchased. Monoclonal anti-Aβ 2D1 antibodies were generated as described in [26]. Rabbit polyclonal antibodies to human Aβ (IBL #18584) and 4G8 (Signet COVANCE), and the APP cytoplasmic domain (Sigma #8717) were purchased.

### Brain lysates, fractionation and immunoblotting

Cerebral cortex, hippocampus and olfactory bulb tissue samples from each hemisphere were homogenized in eight volumes of buffer containing 10 mM Tris-HCl (pH 7.8), 1% (w/v) SDS, 4 M urea, complete protease inhibitor cocktail (Roche-diagnosis), and 1 μM pepstatin on ice with 20 strokes of a Downce homogenizer, sonicated twice for 10 sec, and centrifuged at 15,000 × g for 15 min at 4°C. The supernatant was used for immunoblotting to detect APP, APP CTFs and X11L. The membrane (P100) and cytosolic (S100) fractions were prepared from mouse brain hemisphere samples, including the cerebral cortex, hippocampus and olfactory bulb. The P100 fraction was solubilized as described [11]. This P100 was used to detect APP and APP CTFs, and the S100 was used to detect sAPP in immunoblot analysis. To identify each of the CTFs (C99, C89 and C83) and their respective phosphorylated forms (pC99, pC89 and pC83), samples were subjected to dephosphorylation with λ protein phosphatase as described [27] prior to immunoblotting.

### Quantification of Aβ

Aβ quantification was performed based on the procedure described previously [10]. In brief, cerebral cortex, hippocampus and olfactory bulb samples from each hemisphere were homogenized in four volumes of Tris-buffered saline (20 mM Tris-HCl [pH 7.6], 137 mM NaCl) with 30 strokes of a Downce homogenizer and centrifuged at 200, 000 × g for 20 min at 4°C. The precipitate was further homogenized in nine volumes of TBS with 30 strokes and centrifuged at 100, 000 × g for 20 min at 4°C. One volume of 6 M guanidine chloride in TBS was then added to the precipitate, sonicated for 10 sec twice, and allowed to stand for 1 h at room temperature. The samples were then centrifuged at 130,000 × g for 20 min at 4°C. The supernatant was assayed with sandwich enzyme-linked immunosorbent assay (ELISA) kits (IBL 27714 for human Aβ40 and IBL 27712 for human Aβ42) following dilution with PBS containing 1% (w/v) of BSA and 0.05% (v/v) of Tween 20 as follows: 24-fold for Aβ40; 12-fold for Aβ42 of APP-ibl mice; 500-fold for Aβ40; and 100-fold for Aβ42 of APP23 mice.

### Immunohistochemistry

Frozen mouse brain tissue sections (25-μm thick) were immunostained by either (i) incubation for 1 h in PBS containing 5% (v/v) normal horse serum with mouse monoclonal antibodies, or (ii) incubation for 1 h in PBS containing 5% (v/v) normal goat serum with rabbit polyclonal antibodies for blocking prior to overnight incubation with primary antibody. For Aβ staining, tissue sections were incubated in PBS containing 0.3% (v/v) H_2_O_2 _for 30 min and washed in PBS three times. The sections were then incubated in PBS containing 70% (w/v) formic acid for 1 min prior to blocking. After washing the sections with PBS three times for 10 min, the sections were incubated with horse anti-mouse IgG or goat anti-rabbit IgG antibodies conjugated with biotin (Vector Laboratories), followed by ABC complex. Peroxidase activity was revealed using diaminobenzidine (DAB) as the chromogen. The sections were viewed using a BZ-9000 microscope (KEYENCE, Osaka, Japan).

## Abbreviations

Aβ: β-amyloid; APP: Alzheimer's β-amyloid precursor protein; APPswe; APP carrying Swedish type mutation; sELISA: sandwich enzyme-linked immunosorbent assay; mAPP: mature APP; BACE: β-site cleaving enzyme; PS: presenilin; CTFα: the carboxyl-terminal fragment of APP cleaved at the α-site; CTFβ: the carboxyl-terminal fragment of APP cleaved at the β-site; sAPP: large extracellular N-terminal domain truncated at the α-site (sAPPα) and/or the β-site (sAPPβ); DRM: detergent resistant membrane; X11s: X11 proteins (X11, X11L plus X11L2); X11L: X11-like.

## Competing interests

The authors declare that they have no competing interests.

## Authors' contributions

MK, MS, TM, SH and YS carried out all of the experiments. GI, NT and MM produced the APP-ibl transgenic mouse line. HT, TY and TS participated in the design of the study and the writing of the manuscript. TS conceived the study and was the primary author of the manuscript. All authors read and approved the final manuscript.

## Supplementary Material

Additional file 1 **Supplemental Figures**. Supplemental Figures S1 and S2 Click here for file
