# Supplementary material for: Increased amyloidogenic processing of transgenic human APP in X11-like deficient mouse brain
Source: Mol Neurodegener. 2010 Sep 15;5:35. doi: 10.1186/1750-1326-5-35 (PMC2949864; doi:10.1186/1750-1326-5-35)

Additional file 1

Figure S1. Age-dependent amyloid plaque formation in APP23 mouse brain in the presence or absence of X11L.

Immunostaining of coronal sections of the brain region including the cerebral cortex and hippocampus of APP23 (panels A and C) and APP23/X11L-Ko mice (panels B and D) at 5 (panels A and B) and 9 (panels C and D) months of age is shown. The brain sections were stained with anti-Aβ 82E1 antibody to detect human Aβ. Arrows indicate amyloid plaque. Bar, 50 μm.

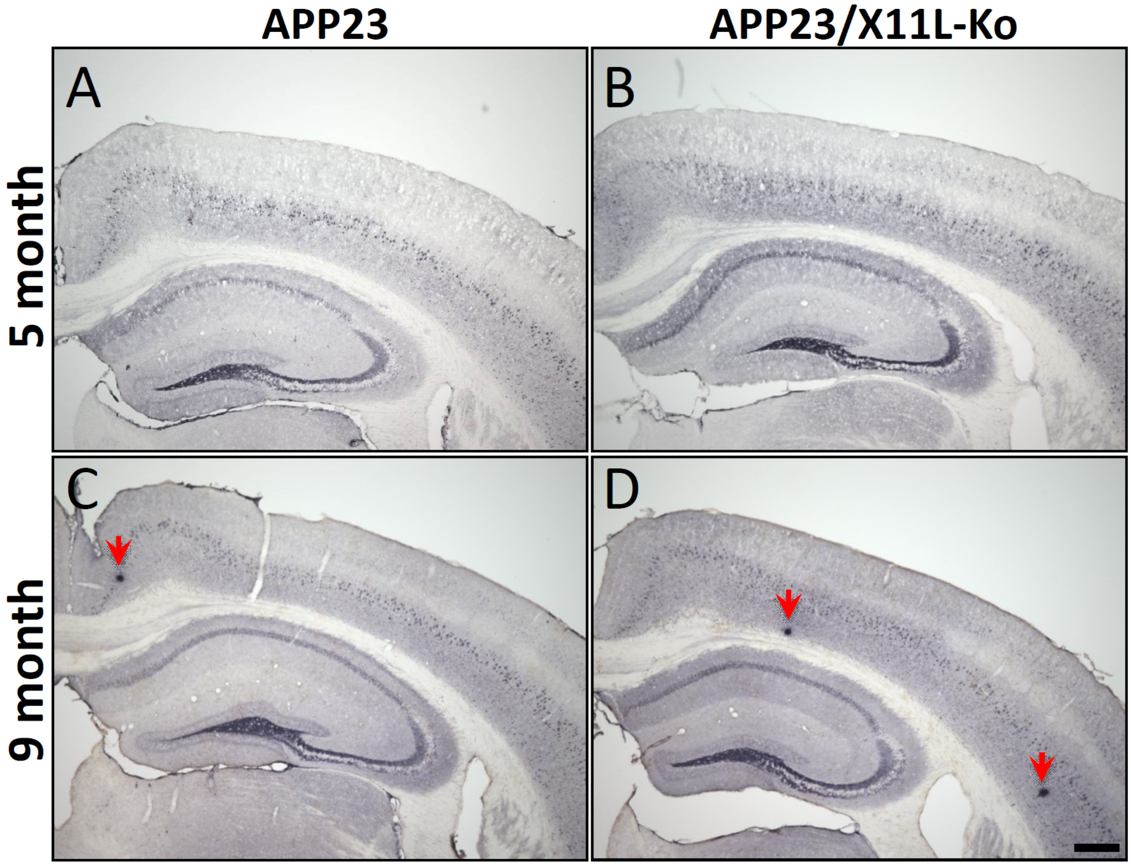

Figure S2. Quantification of amyloid plaques in APP23 mouse brain in the presence or absence of X11L.

Amyloid plaques in the cerebral cortex (CC) and hippocampus (Hip) (panels A and B), and the entorhinal cortex (EC) (panels C and D), of APP23 (panels A and C) and APP23/X11L-Ko mice (panels B and D). The brain sections from 9-month-old mice were immunostained as described in Supplementary Fig. S1. Numbers of plaques within sections (6 to 7 plates in respective regions) with every approximately 0.2-mm interval counted and indicated as numbers per section (panel E). The data were analyzed by Student's t test (n=6 serial sections x 2 individuals and 7 sections x 2 individuals; *, $p<0.05$). The error bars are S.E.

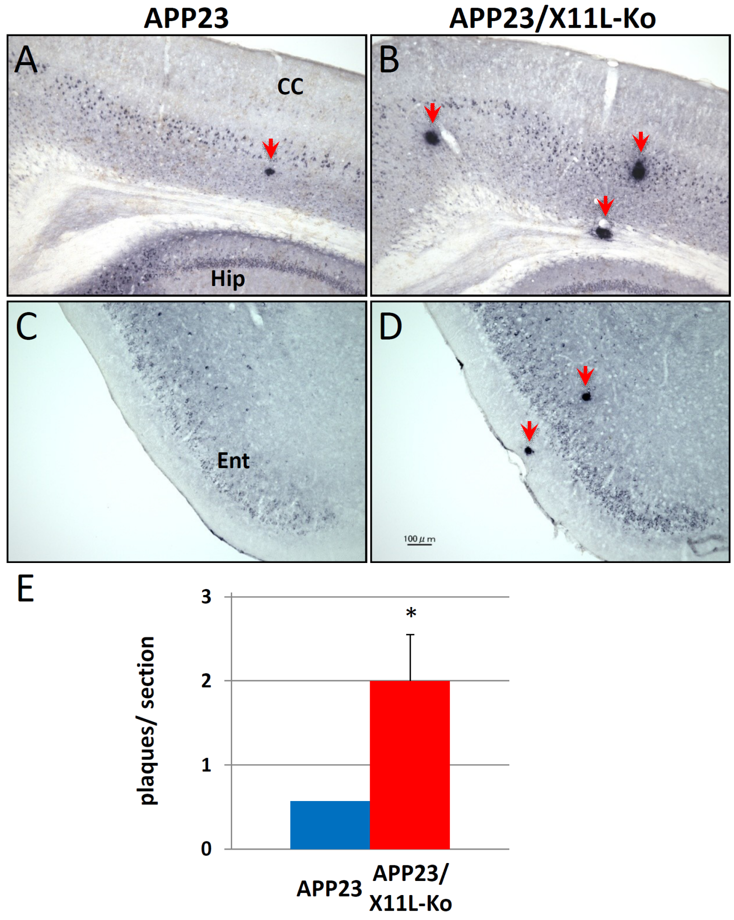

Supplement: Additional file 1 — Supplemental Figures. Supplemental Figures S1 and S2 [file 1750-1326-5-35-S1.PDF]
